# Custom genotyping for substance addiction susceptibility genes in Jordanians of Arab descent

**DOI:** 10.1186/1756-0500-5-497

**Published:** 2012-09-10

**Authors:** Laith N AL-Eitan, Saied A Jaradat, Gary K Hulse, Guan K Tay

**Affiliations:** 1 Centre for Forensic Science, The University of Western Australia, 35 Stirling Highway, Crawley, WA, 6009, Australia; 2 Princess Haya Biotechnology Centre, Jordan University of Science and Technology, Irbid, 22110, Jordan; 3 School of Psychiatry and Clinical Neurosciences, Queen Elizabeth II Medical Centre, The University of Western Australia, Crawley, WA, 6009, Australia; 4 Unit for Research and Education in Alcohol and Drugs, Queen Elizabeth II Medical Centre, The University of Western Australia, Crawley, WA, 6009, Australia

**Keywords:** SNP, *DRD2*, Opiates, Cocaine, Association, Substance addiction, Jordan, Arab

## Abstract

**Background:**

Both environmental and genetic factors contribute to individual susceptibility to initiation of substance use and vulnerability to addiction. Determining genetic risk factors can make an important contribution to understanding the processes leading to addiction. In order to identify gene(s) and mechanisms associated with substance addiction, a custom platform array search for a genetic association in a case/control of homogenous Jordanian Arab population was undertaken. Patients meeting the DSM-VI criteria for substance dependence (*n* = 220) and entering eight week treatment program at two Jordanian Drug Rehabilitation Centres were genotyped. In addition, 240 healthy controls were also genotyped. The sequenom MassARRAY system (iPLEX GOLD) was used to genotype 49 single nucleotide polymorphisms (SNPs) within 8 genes (*DRD1*, *DRD2*, *DRD3*, *DRD4*, *DRD5*, *BDNF*, *SLC6A3* and *COMT*).

**Results:**

This study revealed six new associations involving SNPs within *DRD2* gene on chromosome 11. These six SNPs within the *DRD2* were found to be most strongly associated with substance addiction in the Jordanian Arabic sample. The strongest statistical evidence for these new association signals were from rs1799732 in the C/−C promoter and rs1125394 in A/G intron 1 regions of *DRD2*, with the overall estimate of effects returning an odds ratio of 3.37 (*χ2* (2, *N* = 460) = 21, *p-value* = 0.000026) and 1.78 (*χ2* (2, *N* = 460) = 8, *p-value* = 0.001), respectively. It has been suggested that *DRD2*, dopamine receptor D2, plays an important role in dopamine secretion and the signal pathways of dopaminergic reward and drug addiction.

**Conclusion:**

This study is the first to show a genetic link to substance addiction in a Jordanian population of Arab descent. These findings may contribute to our understanding of drug addiction mechanisms in Middle Eastern populations and how to manage or dictate therapy for individuals. Comparative analysis with different ethnic groups could assist further improving our understanding of these mechanisms.

## Background

Substance addiction and dependency has been influenced by both genetic and environmental risk factors [1]. It has been estimated that genetic factors contribute to 40%–60% of the vulnerability to drug addiction, and environmental factors provide the remainder [2-6]. However, there is also evidence for shared genetic vulnerability to two or more drugs such as cannabis, sedatives, stimulants and opiates which may explain the finding that addicted patients are often dependent on more than one category of drug [2-9]. The presence of unique and shared genetic factors for substance addiction [5,7] leads to the hypothesis that there is an association between specific genetic polymorphisms and increased risk of substance addiction.

Genetic susceptibility to addiction is the result of the interaction of many genes related to the central nervous system (CNS) [9-12]. In this system, dopamine is thought to be the primary neurotransmitter involved in the mechanisms of reward and reinforcement [13-16]. The function of dopamine is mediated by two classes of dopamine receptors termed D_1_ like and D_2_ like families. The D_1_ like family (D_1_ and D_5_ dopamine receptors) mediate a reduction in the drive to seek reinforcement effects, in contrast to the family of D2-like receptors (including D_2_, D_3_, and D_4_ dopamine receptors) mediate both reward and reinforcement effects [6,15-19]. The dopamine receptor gene family, which comprises *DRD1* (MIM *126449), *DRD2* (MIM *126450), *DRD3* (MIM *126451), *DRD4* (MIM * 126452) and *DRD5* (MIM *126453) is a prime candidate gene family for influencing substance abuse because this gene family is thought to play one of the most important roles in the neurobehavioral signaling pathways implicated in substance addiction [15,18].

Several studies have implicated a role for the products of dopamine receptor gene variants in mediating the behavioral and neurochemical properties of opiates such as heroin [8,16]. It has also been suggested that the endogenous dopamine system may also contribute to the development of dependence on other drugs of abuse such as alcohol, cannabis, cocaine and amphetamines [7,11,12]. Various studies have shown that dopamine receptors are involved in reinforcement of drug use in addicted individuals [15]. Other neurotransmitters are also thought to play a role in reinforcement including the dopamine active transporter (*DAT*; gene symbol *SLC6A3*, MIM *126455) [20], neurotrophines such as Brain-derived neurotrophic factor (gene symbol *BDNF*, MIM *113505) [21-24] and enzymes systems such as catechol-O-methyltransferase (gene symbol *COMT*, MIM *116790) [25]. All of these genes are expressed within the meso-corticolimibic dopamine system or associated structures such as the nucleus accumbens, ventral tegmental area, amygdala, prefrontal cortex, hippocampus and cerebral cortex [5,11,12,21].

Human molecular genetic studies are also implicating the dopamine receptor gene family in substance use disorders. The rs5326 SNP is located in the 5'-UTR of *DRD1* gene and has been linked to heroin dependence in African Americans [26]. While there are no similar confirmed associations between *DRD2* gene and substance addiction [15,27], some variants within *DRD2* gene such as the rs1799732 SNP (C/-C, 5'-UTR) warrant further investigation as these variants have a functional effect on gene expression [28]. The *DRD3* gene has been reported to be associated with substance abuse [29] and cocaine [30] and heroin abuse [31] but others have not reported association with abuse of either drug [32,33]. The rs3758653 SNP located in the 5'-UTR of the *DRD4* gene has been reported to be associated with heroin dependence in 53 heroin Hungarian addicts [34]. The *DRD5* gene has not been the subject of many genetic studies.

The dopamine active transporter (*DATSLC6A3*) is widely distributed throughout the brain in areas of dopaminergic activity [20]. The DA transporter *DAT1* mediates the active reuptake of *DA* from the synapse and is a principal regulator of dopaminergic neurotransmission. Its addictive effects are thought to be principally mediated through blockage of *DAT,* resulting in a substantial increase in the concentration of extracellular *DA* and stimulation of neurons in brain regions involved in reward and reinforcement behavior [35]. Family and twin studies suggest that *DAT1* is a substantial genetic factor in the vulnerability of individuals to cocaine dependence after exposure [36-38]. Therefore, polymorphic functional variants in the *DAT* gene may act to modify susceptibility to substance abuse and dependence.

Brain-derived neurotrophic factor (*BDNF*) is a member of the nerve growth factor family. This family is a group of structurally related secretory proteins widely expressed in neurons and their target cells [39]. Induced by cortical neurons, *BDNF* is required to support existing neurons in the brain and help in the growth and differentiation of new neurons and synapses [40-42]. Studies in animals and humans suggest that *BDNF* influences the dopaminergic and serotonergic functions that are heavily linked to substance addiction [43-47]. In mice, *BDNF* administration or *BDNF* genetic knockouts have shown that this factor can alter drug preference or drug-induced behavior. In humans, Uhl et al. (2001) used 1494 SNPs to scan for vulnerability genes for polysubstance abuse. Using 1004 European American and African American samples; they found that positive association markers flank the *BDNF* gene and Val66Met at rs6265 position was associated with drug addiction vulnerability [46]. Recently, various studies have shown that the Val66Met substitution in the prodomain may affect intracellular trafficking and activity-dependent secretion of *BDNF*[47,48]. Overall these animal and human studies indicate that *BDNF* may be involved in the mechanisms underlying substance addiction [49].

Catechol-O-methyltransferase (*COMT*) is one of several enzymes that metabolises catecholamine such as dopamine, epinephrine and norepinephrine and play a role in the reinforcement mechanism [5,7]. Nikoshkov et al. (2008) suggests that heroin addicts with homozygous genotype at position rs4680 Met158/Met158 have a significant up-regulation of *COMT* gene expression [50]. In contrast, heroin addicts with the heterozygous genotype (Vall158/Met158) or homozygous genotype of Vall158 at this position show a down-regulation of *COMT* gene expression. Goldman (2005) reported that the Val158 variant catabolizes dopamine up to four times the rate of its methionine counterpart, resulting in significant lower synaptic dopamine levels following neurotransmitter release. This ultimately reduces dopaminergic stimulation of the post-synaptic neuron [5]. Therefore, due to the role of *COMT* in prefrontal dopamine degradation, the Val158Met polymorphism is thought to be associated with increased risk of substance addiction by modulating dopamine signaling in the frontal lobes.

In the present study, we examined 49 SNPs within eight candidate genes, the dopamine receptors *DRD1*, *DRD2*, *DRD3*, *DRD4* and *DRD5*, the dopamine transporter (*SLC6A3*) brain-derived neurotrophic factor (*BDNF*) and catechol-O-methyltransferase (*COMT*) for genetic association analysis with substance addiction in Arab individuals. To the best of our knowledge, this report is the first genetic association study for substance addiction in a Middle Eastern population of Arab descent. These findings may prove crucial to our understanding of substance addiction mechanisms in Arab populations. At the individual level, this knowledge may improve patient management and treatment.

## Methods

### Subjects

All substance addiction subjects were recruited from the National Centre for Rehabilitation of Addicts (NCRA) at Jordanian Ministry of Health and the Drug Rehabilitation Centre at the Jordanian Public Security Directorate (DRC-PSD). They were diagnosed as having substance addiction using DSM-IV criteria (American Psychiatric Association, 1994) [51]. A semi-structured interview based on the Addiction Severity Index (ASI) criteria [52] was used to collect clinical and demographic data for each subject. The clinical and demographic data were collected by an administering officer from each of the addiction treatment Centres. The clinical data included current drug of abuse, age at first use of drug, onset and years of drug use, substance and psychiatric treatment, drug overdose and history of substance abuse. Demographic data collected included date of birth, gender, nationality was also provided. All data was coded and no specific individual was identified. The mean age (±*SD*) of these subjects was 32.7 (±8.4) years with an age range of 18 to 58 years.

In addition, 240 healthy males from an ethnically homogenous Jordanian Arab population with no lifetime history of psychosis or mood disorders, or alcohol or heroin dependence according to the DSM-IV, were used as controls. These controls were recruited from the Blood Bank of the King Abdullah Hospital University, Jordan University of Science and Technology. These controls were frequency matched by age, sex and ethnicity to the case subjects. The mean age (±*SD*) of the controls was 31.5 (± 5.6) years with an age range of 18 years to 54 years.

This study was conducted according to the provisions of the Australian Medical Association Code of Ethics (2006) and the World Medical Association Declaration of Helsinki (World Medical Association, 2008). The study was also subject to, and in compliance with, the National Statement on Ethical Conduct in Human Research, Australia (2007). Ethical approval to conduct this research was granted by the Human Research Ethics Committee of The University of Western Australia (Ref No. RA/4/1/4344). This study was also approved by the Human Ethics Committee of the Jordanian Ministry of Health (Ref No. Development/Trainees/535) and by the Institutional Review Board of the Jordan University of Science and Technology (Ref No. RA/16/1/2010). Written informed consent was obtained from all subjects and controls before participation in the study.

### DNA extraction

After blood was drawn into EDTA tubes, genomic DNA ^®^ Blood Kit (Qiagen, Valencia, CA, USA) according to the recommendations of the manufacturer. Briefly, 300 μl of whole blood from each sample was mixed with 200 μl of lysis buffer (50 mM Tris pH 8.0, 100 mM EDTA, 100 mM NaCl, 1% SDS) and 40 μl of Proteinase K. 100 μl of isopropanol and 500 μl of Inhibitor Removal Buffer (5 M guanidine-HCl, 20 mM Tris–HCl pH 6.6) was then added. The DNA was washed with a buffer (20 mM NaCl; 2 mM Tris- HCl; pH 7.5) and centrifuged twice at 2,000 rpm. The DNA was washed using cold 70% ethanol, centrifuged at 3,000 rpm and the supernatant was discarded, leaving a pellet that contained purified genomic DNA. The DNA pellet was diluted in TE buffer (1 mM EDTA; 10 mM Tris–HCl, pH 7.5) to a concentration of approximately 50 ng.μl-1. DNA concentration (ng/μl) and purity (*A*_260/280_) were also verified using the Nano-Drop ND-1000 UV–vis Spectrophotometer (NanoDrop Technology, Wilmington, DE) and subsequently adjusted to approximately 100 ng/μL. Purified DNA was stored at −80°C before use.

### Genotyping

In this study, 49 single nucleotide polymorphisms (SNPs) within eight genes (*DRD1*, *DRD2*, *DRD3*, *DRD4*, *DRD5*, *BDNF*, *SLC6A3* and *COMT*) were selected from public databases including the SNP database of the National Centre for Biotechnology Information (NCBI; http://www.ncbi.nlm.nih.gov/SNP/), the Applied Biosystems SNP database (http://www.appliedbiosystems.com) and the International HapMap Project (http://www.hapmap.org/). The positions of the SNPs in these selected genes and the relative distance to the translation initiation site are given in Table 1.

**Table 1:** List of Genes, their SNPs and positions, and genotyping data based on the whole cohort (460 subjects)

**Gene**	**Gene location**	**SNP _ID**	**Position**^**a**^	**SNP**	**SNP location**	**Discrepancy rate**^**b**^	**Call rate**^**c**^
*DRD1*	5q35.1	rs5326	174802802	G > A	5'-UTR	0.25%	99%
*DRD2*	11q23	rs1800496	112788698	C > T	Exon 7	0.00%	100%
		rs6277	112788669	T > C	3'-UTR	0.15%	99%
		rs2511521	112790509	T > C	Intron 4	0.00%	100%
		rs12574471	112821446	C > T	Intron 1	0.00%	100%
		rs2283265	112790746	G > T	Intron 4	0.00%	100%
		rs6279	112786283	C > G	3'-UTR	0.00%	100%
		rs4581480	112829684	T > C	5'-UTR	0.00%	100%
		rs4350392	112840927	C > A	5'-UTR	0.00%	100%
		rs10891556	112857971	G > T	5'-UTR	0.00%	100%
		rs7103679	112808884	C > T	Intron 1	0.00%	100%
		rs4938019	112846601	T > C	Intron 1	0.00%	100%
		rs1076560	112788898	G > T	Intron 5	0.00%	100%
		rs2075654	112794276	G > A	Intron 2	0.00%	100%
		rs7125415	112815891	C > T	5'-UTR	0.00%	100%
		rs4648317	112836742	C > T	Intron 1	0.00%	100%
		rs1125394	112802395	A > G	Intron 1	0.00%	100%
		rs4648318	112818599	A > G	Intron 1	0.00%	100%
		rs12363125	112791126	A > G	Intron 5	0.00%	100%
		rs2734836	112796449	G > A	Intron 2	0.05%	99%
		rs12364283	112852165	T > C	5'-UTR	0.00%	100%
		rs1799978	112851561	A > G	5'-UTR	0.00%	100%
		rs6275	112788687	C > T	Exon 7	0.15%	99%
		rs1800497	112776038	C > T	Exon 8	0.00%	100%
		rs1079597	112801496	A > G	Intron 1	0.00%	100%
		rs1799732	112851462	-C	5'-UTR	0.00%	100%
		rs1800498	112796798	C > T	Intron 2	0.00%	100%
*DRD3*	3q13.3	rs6280	115373505	C > T	Exon 1	0.07%	99%
*DRD4*	11p15.5	rs3758653	626399	C > T	5'-UTR	0.05%	99%
*DRD5*	4p16.1	rs10033951	9388678	C > T	5'-UTR	0.05%	99%
*SLC6A3*	5p15.3	rs2963238	1497427	A > C	Intron 1	0.12%	99%
		rs6876225	1459036	C > A	Intron 11	0.00%	100%
		rs11564773	1449813	A > G	Intron 14	0.00%	100%
		rs1042098	1447815	T > C	3'-UTR	0.15%	99%
*BDNF1*	11p13	rs7103873	27656893	C > G	Intron 1	0.07%	99%
		rs1401635	27650567	C > G	Intron 1	0.00%	100%
		rs11030102	27638172	C > G	Intron 1	0.00%	100%
		rs17309930	27705069	A > C,G > T	Intron 1	0.07%	99%
		rs6265	27636492	G > A	3'-UTR	0.00%	100%
*COMT*	22q11.21	rs737866	18310109	T > C	5'-UTR	0.00%	100%
		rs4680	18331271	A > G	Exon 2	0.00%	100%

^*a.*^Chromosome positions are based on NCBI Human Genome Assembly Build 36.3.

^*b.*^Ratio of the number of discordant genotypes to the number of duplicates.

^*c.*^Ratio of the number of valid genotypes to the number of subjects genotyped (*N* = 460) at each locus.

**Figure 1:**
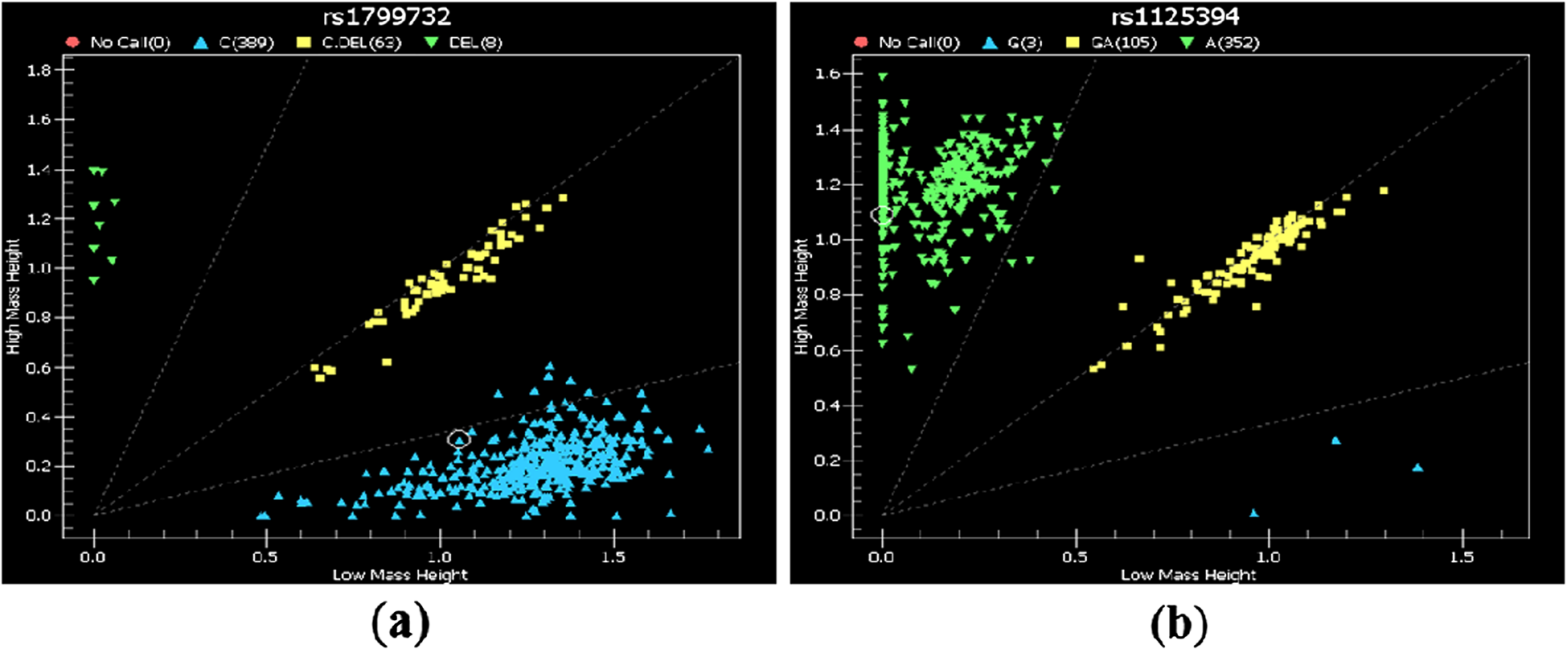
**Representative Scatter plot from sequenom data.** The left panel (**a**) and right panel (**b**) illustrate the scatter plot of rs1799732and rs1125394 SNPs within *DRD2* gene, respectively. These two SNPs showed the strongest statistical evidence for association with substance addiction in Arab population. The X and Y axes on both plots denote the mass height measurement for the two alleles (C, C.DEL, low mass allele versus high mass allele) at the rs1799732 SNP (panel a) and for the two alleles (G, A, low mass allele versus high mass allele) at the rs1125394 SNP (panel b). Each point represents the measurements for a single individual. The points in the both panels are colored according to the genotype calls. For example in the left panel (a), green color denotes –C genotype; yellow color denotes C/-C genotype and blue color denotes CC genotype and red color denotes no call. Genotypes determined by sequenom MassARRAY^®^ system (iPLEX GOLD) for all 49 SNPs were highly accurate with average success rate 100%. Genotype discrepancy average (±*SD*) rate across the 49 loci were only 0.02% (±0.06%) in the whole cohort (460 subjects)

SNP genotyping using the sequenom MassARRAY^®^ system (iPLEX GOLD) (Sequenom, San Diego, CA, USA) was performed according to the manufacturer’s recommendations (Sequenom, San Diego, CA, USA). Briefly, PCR and single base extension primers (SBE) were designed using MassARRAY assay design 3.1 software (Sequenom MassARRAY system) that allows iPLEX reactions for SBE designs with the modified masses associated with the termination mix. Manufacturer’s instructions for the multiplex reaction were followed in the whole process, including the PCR amplification (Sequenom, San Diego, CA, USA), the shrimp alkaline phosphatase (SAP) enzyme (Sequenom, San Diego, CA, USA) treatment to dephosphorylate dNTPs unincorporated in the PCR, the SBE reactions using an iPLEX GOLD assay (Sequenom, San Diego, CA, USA), and the clean-up with a resin kit (Sequenom, San Diego, CA, USA) to desalt the iPLEX reaction products. PCR and SBE primers sequences and all protocol conditions are available upon request. Reaction products were dispensed onto a 384-element SpectroCHIP bioarray (Sequenom) using a MassARRAY nanodispenser and assayed on the MassARRAY platform. Mass differences were detected with matrix-assisted laser desorption/ionization time-of-flight mass spectrometry (MALDI-TOF MS). MassARRAY Workstation v.3.3 software was used to process and analyse the iPLEX SpectroCHIP bioarray. Typer Analyzer v.4.0.2 software was used to analyse all genotypes obtained from the assays. Scatter plots of rs1125394 and rs1799732 SNPs within *DRD2* gene were colored according to genotype calls: AA (green), GA (yellow) GG (blue) and no call (red) (Figure 1).

### Quality control (QC)

Data cleaning was performed using the PLINK software developed by Purcell et al. (2007) [49]. Using this software, the genotype results of each marker are accepted only if the success rate is at least 90%. SNPs were excluded from the analysis based on the following criteria: (1) minor allele frequency (MAF) < 0.05, or (2) missingness per SNP > 5%, or (3) significant deviation (*p-value* < 1.0 E-06) from the Hardy-Weinberg equilibrium (HWE).

### Statistical methods

#### Hardy-Weinberg equilibrium

The HWE provides a measure of wether an evolutionary event has influenced an allele frequency. Theoretically calculated, expected and seen genotype frequencies are compared to each other and a Pearson χ2 test is used to test whether they significantly differ from each other. In this study, the estimated genotype frequencies were calculated as follows: *p*2 + 2*pq* + *q*2 = 1, in which *p* represents the frequency of one allele, and *q* represents the frequency of the other allele [53]. Significant deviations from HWE may indicate genotyping errors.

#### Genetic association analysis

The software package PLINK [54] was used to test for association between genetic variants and substance addiction.

#### Correction for multiple testing

In order to correct for the effect of multiple testing for a given phenotype, the effective number of SNPs using the method of Li and Ji (2005) was estimated [55], which employs a modification of an earlier approach by Nyholt (2004) [56]. After obtaining the effective number of SNPs (*N*_*em*_), a modified Bonferroni procedure was applied to identify a target alpha level (0.05/ *N*_*em*_) that would maintain an overall significance level of 0.05 or less.

## Results

### The study sample

Altogether 460 individuals were analysed in this study to identify potential candidate genes related to substance addiction. The study sample consisted of 220 Jordanian Arab individuals with substance addiction and 240 matched controls. The average age (±*SD*) was 32.70 (± 8.4) and 31.5 (± 5.6) years, respectively. No drug dependent individual or control had any psychiatric diseases according to the DSM-IV criteria assessment. There were no significant differences found between individuals with drug dependence and controls with regard to age and sex. Clinical and demographic data including gender, age, current drug abuse, dependence variables, drug overdose or toxicity, history of drug use and substance and psychiatric treatment is given in Table 2.

**Table 2:** Characteristics of 220 substance abuse patients of Arab origin in this study

**Category**		**Subcategory**	**Value (n)**	**Percentage (%)/Mean ± SD**
Demographic data	Gender	Male	220	100.0%
		Female	0	0.0%
	Age (years)	18-20	12	5.5%
		21-39	165	75.0%
		+40	43	19.5%
Drug/alcohol problem	Current drug abuse	Nicotine	203	92.0%
		Opiates	185	84.0%
		Cannabis	128	58.0%
		Alcohol	117	53.0%
		Amphetamine	31	14.0%
		Cocaine	7	3.0%
	Dependence	Age first drug use (years)	220	18.7 ± 10.1
		Age of onset (years)	220	20.3 ± 10.9
		Duration (years)	220	7.6 ± 6.6
		Frequency (days/week)	220	3 ± 1.5
	Drug overdose		100	45.5%
	History of drug use		53	24.0%
Previous treatment	Substance treatment	Alcohol	117	53.0%
		Drugs	185	84.0%
	Psychiatric treatment	Inpatient	15	7.0%
		Outpatient	20	9.0%

Mean (*M*) data are provided with ± Standard Deviation (*SD*).

### HWE test

HWE tests were performed in case and control groups for the studied polymorphisms respectively. All polymorphism were in HWE in both case and control groups except for the three SNPs within *DRD2* gene (rs1801028, rs2734838, and rs1110976) and two SNPs within *SLC6A3* gene (rs27048, rs6347). Two SNPs for *COMT* gene (rs1544325, rs2239393) had *p-values* < 1.0 E-06 and were excluded from the study.

### Quality control (QC)

All the genotyped SNPs were checked for HWE and Mendelian errors. All duplicates were identical, water controls were clean, markers were in HWE and no Mendelian errors were observed. Genotypes determined by sequenom MassARRAY^®^ system (iPLEX GOLD) for all 49 SNPs were highly accurate with an average success rate of 100%. The genotype discrepancy average (±*SD*) rate across the 49 loci was only 0.02% (±0.06%) in the whole cohort (460 subjects).

### The candidate genes and the SNPs

The NCBI, dbSNP and HapMap databases were used for the SNP selection. The goal was to select SNPs that had significant functional relevance, covered the genes of interest as widely as possible, and had been previously genotyped. Using these criteria, a total of 49 SNPs were selected (Table 2). Of these, 41 (82%) passed quality control and were used in the association analysis.

### Association of SNPs candidate genes with substance addiction

Association analysis of eight genes (*DRD1 *,*DRD2 *,*DRD3 *,*DRD4 *,*DRD5 *,*SLC6A3 *,*BDNF* and *COMT*) with substance addiction was performed using PLINK Software [54]. The association *p- values* from the PLINK genetic association analysis are shown in Table 3. 

**Table 3:** Association of genes SNPs with opiate drug dependence

**Gene**	**SNP _ID**	**SNP**	**M_A**^**a**^	**F_A**^**b**^	**F_U**^**c**^	**Pearson chi-square**	***p-value***^**d**^	**OR CMH**^**e**^
*DRD1*	rs5326	G > A	A	0.172	0.144	1.32	0.256	0.256
*DRD2*	rs1800496	C > T	T	0.027	0.015	1.86	0.173	1.903
	rs6277	T > C	T	0.427	0.460	1.05	0.307	0.873
	rs2511521	T > C	G	0.370	0.383	0.16	0.685	0.946
	rs12574471	C > T	T	0.196	0.161	1.85	0.173	1.265
	rs2283265	G > T	T	0.146	0.087	8.70	**0.001**	2.785
	rs6279	C > G	C	0.406	0.435	0.79	0.374	0.888
	rs4581480	T > C	C	0.095	0.075	1.29	0.256	1.308
	rs4350392	C > A	A	0.173	0.217	2.82	0.093	0.755
	rs10891556	G > T	T	0.187	0.235	3.18	0.074	0.748
	rs7103679	C > T	T	0.121	0.083	3.48	0.062	1.506
	rs4938019	T > C	C	0.170	0.216	3.13	0.076	0.743
	rs1076560	G > T	T	0.157	0.108	4.73	**0.031**	1.531
	rs2075654	G > A	A	0.120	0.075	5.43	**0.021**	1.689
	rs7125415	C > T	T	0.100	0.118	0.78	0.376	0.829
	rs4648317	C > T	T	0.170	0.216	3.12	0.076	0.743
	rs1125394	A > G	G	0.152	0.091	8.00	**0.001**	1.780
	rs4648318	A > G	G	0.379	0.361	0.29	0.593	1.080
	rs12363125	A > G	C	0.513	0.472	1.52	0.217	1.170
	rs2734836	G > A	A	0.146	0.090	7.00	**0.001**	1.720
	rs12364283	T > C	G	0.097	0.129	2.24	0.134	0.730
	rs1799978	A > G	G	0.141	0.110	2.03	0.154	1.330
	rs6275	C > T	T	0.404	0.429	0.60	0.440	0.900
	rs1800497	C > T	T	0.195	0.156	2.37	0.123	1.310
	rs1799732	-C	-C	0.146	0.067	21.00	**0.260E-4**	3.370
	rs1800498	C > T	C	0.509	0.466	1.66	0.190	1.180
*DRD3*	rs6280	C > T	C	0.355	0.370	0.21	0.645	0.938
*DRD4*	rs3758653	C > T	C	0.263	0.287	0.66	0.410	0.886
*DRD5*	rs10033951	C > T	T	0.306	0.325	0.36	0.550	0.917
*SLC6A3*	rs2963238	A > C	A	0.436	0.462	0.63	0.427	0.899
	rs6876225	C > A	A	0.027	0.035	0.49	0.480	0.764
	rs11564773	A > G	G	0.043	0.437	0.00	0.978	0.991
	rs1042098	T > C	C	0.363	0.342	0.42	0.515	1.094
*BDNF1*	rs7103873	C > G	C	0.463	0.510	2.02	0.154	0.827
	rs1401635	C > G	C	0.231	0.212	0.50	0.481	1.118
	rs11030102	C > G	G	0.163	0.138	1.17	0.279	1.221
	rs17309930	A > C,G > T	A	0.136	0.127	0.17	0.679	1.084
	rs6265	G > A	A	0.182	0.161	0.748	0.387	1.164
*COMT*	rs737866	T > C	C	0.363	0.312	2.69	0.100	1.259
	rs4680	A > G	A	0.475	0.502	0.67	0.411	0.897

^*a.*^M_A: minor allele for whole cohort sample.

^*b.*^F_A: minor allele frequency in affected individuals (substance addiction cases).

^*c.*^F_U: minor allele frequency in unaffected individuals (healthy controls).

^*d.*^*p-value*: two tailed *p-value* from the 2x2 allele count chi-squared test; *p* <0.05 (Bonferroni-adjusted).

^*e.*^OR CMH: allelic odds ratio from the 2x2xK Cochran-Mantel-Haenszel’s test.

#### Dopamine receptor genes

The top scoring SNPs for association with substance addiction were from the *DRD2* gene (Figure 1). The significant *p-values* for genotypic frequency ranged from 0.03 to 0.000026 for six SNPs within *DRD2* gene on chromosome 6 (Table 3). The strongest statistical evidence for these new association signals were from rs1799732 in the C/−C promoter and rs1125394 in A/G intron 1 regions of *DRD2*, with the overall estimate of effects returning an odds ratio of 3.37 (*χ2* (2, *N* = 460) = 21, *p-value* = 0.000026) and 1.78 (*χ2* (2, *N* = 460) = 8, *p-value* = 0.001), respectively. The *p-values* for allelic frequency ranged from 0.01 to 0.0001 for five SNPs (rs2283265 (G/T, intron 4), rs1125394 (A/G, rs2075654, intron 1), rs2734836 (G/A, intron 2), and rs1799732 (C/-C, 5'-UTR) (data not shown) within *DRD2* gene. The strongest statistical evidence of allelic frequency for these new association signals were from rs1799732 (*χ2* (1, *N* = 460) = 15, *p-value* = 0.0001) and rs2283265 (*χ2* (1, *N* = 460) = 8, *p-value* = 0.005).

#### Solute carrier family 6, member 3(SLC6A3), brain-derived neurotrophic factor (BDNF) and catechol-O-methyltransferase (COMT) genes

There were no significant difference of genotype (Table 3) or allele frequencies (data not shown) of the studied SNPs in the *SLC6A3*, *BDNF* and *COMT* genes between subjects with substance addiction and normal controls.

## Discussion

Although epidemiologic studies have shown that substance addiction is strongly influenced by genetic factors, the number and identity of vulnerability genes remain unknown [1,3-8]. This is the first study to examine eight candidate genes for association with substance addiction in individuals of Arab descent. These eight genes were the Dopamine receptors (*DRD1 *,*DRD2 *,*DRD2 *,*DRD3* and *DRD5*), Solute Carrier Family 6, Member 3 (*SLC6A3*), Brain-Derived Neurotrophic Factor (*BDNF*) and Catechol-O-Methyltransferase (*COMT*). Altogether 460 individuals were genotyped using 49 SNPs from these eight genes. Of the samples tested, 220 were from substance addicted male subjects of Arab descent. The control group were an ethnically homogenous Jordanian Arab population with no lifetime history of psychosis, mood disorders or substance dependence.

Both dopamine and non-dopamine neurochemical pathways through neurotransmitters (*SLC6A3*), neurotrophic factors (*BDNF*) and enzymes (*COMT*) are influenced by drugs and their psychoactive and addictive effects [8,10,12]. Dopamine is one of the main neurotransmitters involved in the stimulation of reward pathways, which is the important feature of substance addiction [13,15,57]. It has been suggested that dopamine receptor genes play a role in the genetics of substance addiction [58-60]. Previous studies have emphasized the importance of dopamine gene family specifically *DRD2* gene as a general risk factor for substance dependence rather than a marker of risk for a particular drug [13,15,18]. However, various genetic association studies reported that there are inconsistencies in the frequency of alleles within *DRD2* gene in different populations. For example, Barr and Kidd reported that the A1 allele frequency differs dramatically among the population studied from as low as 0.09 to as high as 0.075 [61].

As many studies indicated that multiple substances influence dopaminergic system activity, the investigation of substance addiction may result in a complete examination of gene risk [7,11,12]. In this study, none of the polymorphisms within the eight genes differed significantly for allele or genotype frequencies, with exception of six polymorphisms (rs2283265, rs10765560, rs2075654, rs1125394, rs2734836 and rs1799732) within the *DRD2* gene. The strongest statistical evidence for these association signals was found within the *DRD2* gene at two sites: rs1799732 (C/-C, 5'-UTR) and rs1125394 (A/G, intron 1). The strongest evidence of allelic frequency for these association signals were from rs1799732.

The rs1799732 (C/-C, 5'-UTR) is of particular interest because there is evidence that this allele has a functional effect on *DRD2* gene expression [27]. The dopaminergic system is involved in reward and reinforcing mechanisms in the brain [13,57] specifically the positive reinforcing effects of substance addiction [59]. Animal and human studies of addiction indicate that *DRD2* plays a critical role in the mechanism of reward and reinforcement behavior [60-63]. Various animal studies reported that opiate rewarding effects were absent in mice lacking D2 receptors, while *DRD2* overexpression in transgenic mice led to reduced self-administration of alcohol [60,62]. A positron emission tomography study of human brain showed that D2 receptor density in the brain decreased significantly in alcoholic compared with control subjects [63,64]. These findings suggest that genetically determined variation in *DRD2* expression and function can alter reward responses to a variety of substances and may contribute to vulnerability to heroin dependence in humans. For example, *DRD2* gene was previously studied by Xu et al. (2004) to examine the susceptibility of this gene with heroin dependence in Chinese and German population [65]. This study found that genetic polymorphisms, specifically rs1799732 (C/-C), within *DRD2* gene play a role as a susceptibility gene with heroin dependence in Chinese but not in German population [65].

Association with substance addiction was not seen in the studied SNPs within *SLC6A3BDNF* and *COMT* genes. Conflicting results have been published in various studies on the influence of these genes on the increased risk of substance addiction [35-37,42-46]. Candidate gene analysis is problematic because the prior probability of seeing true association is exceptionally low [66], unless a very strong case of specific phenotype for involvement of a particular gene can be made. This is not applied to substance addiction because compelling biological evidence implicating particular neurotransmitter receptors in addiction is absent, with the possible exception of the opioid receptor gene family, and prior probability is impossible to determine [58]. Thus *p-values* of 0.05 are more likely to be chance occurrences, especially when using cases and controls where hidden population stratification as confounding factor is an inherent danger. However, a risk of population stratification as a confounding factor was not found in this study because the Jordanian Arab population are considered to be genetically homogenous population. This offers an advantage for genetic studies. For example, the numbers of different variations in the genes behind phenotypes are expected to be smaller than in more heterogeneous populations. This increases the probability to find genetic associations [67]. Therefore, even a small study sample from a genetically homogenous population, like the sample of subjects used in this study, can give accurate results.

In this study, genotyping was carried out by sequenom MassARRAY^®^ system for 49 SNPs. The NCBI, dbSNP, HapMap databases and previous published data were used to select the studied SNPs, yielding reliable candidate SNPs database for genetic association analysis. In this array we focused on genes of particular interest for drug, alcohol and neuropsychiatric researchers because they were reported to be involved in drug dependence and other neurological and psychiatric disorders [4-12]. The chosen SNPs were also selected because they showed the greatest potential to distinguish between substance addicts’ individuals and control subjects in previous studies [4-12]. Therefore, the distribution of SNPs through the selected genes was optimal.

Various studies showed a risk of false positive results due to population stratification. However, a risk of false positive results was not found in this study because genotypic frequencies of chosen SNPs in the patients and controls met HWE expectations. In addition, it is likely that there were genotyping errors. However, genotyping errors were minimized by genotyped each patient twice in order to avoid technical errors as evidenced by the low average rate of genotype discrepancy. Genotyping was conducted for patients under the same conditions and during the same period. Genotypes were also evaluated by investigators who were blind to the status of the subject and any discrepancies were resolved by test replication.

A confounding factor which could have contributed to the observed variations in the between this study and previous studies is the heterogeneity of population based on gender [66,67]. However in our study, only male individuals with substance addiction were genotyped. Therefore, the generalisation of the results to all substance addicts’ individuals is limited. Another confounding factor is differences in phenotype in addiction such as polysubstance use, severity of addiction and the use of unstructured clinical interviews to obtain phenotypic data could affect the genetic association analysis. However, these confounding factors are not found in our study as a specific clinical structural interview was designed based on the DSM-IV criteria and the Addiction Severity Index (ASI) for collecting clinical and phenotypic data [52]. The careful and extensive interview based phenotypic data collection has been performed by highly trained psychiatrist consultants, yielding exceptionally reliable phenotype data. In addition, the study sample is strongly enriched with regular substance addicts’ individuals giving more statistical power.

## Conclusion

Overall our results indicate that the *DRD1 *,*DRD3 *,*DRD4 *,*DRD5 *,*SLC6A3 *,*BDNF* and *COMT* genes are not likely to be a major genetic risk factor for substance addiction in the Arab population, with the exception of strong association between substance addiction and *DRD2* gene. However, it has been proposed that defects in various combinations of these genes for these neurotransmitters results in the Reward Deficiency Syndrome (RSD) and that indivuals at risk for abuse of the unnatural rewards [68]. Because of its importance, *DRD2* gene was a major candidate gene [68-70]. Several studies in the past decade have shown that in various subject groups the *DRD2* gene is associated with alcoholism, drug abuse, smoking, obesity, compulsive gambling, and several personality traits [69,70]. A range of other dopamine, opioid, cannabinoid, norepinephrine, and related genes have since been considered to be candidate genes. Like other behavioral disorders, these genes are polygenically inherited and each gene accounts for only a small per cent of the variance [68,69]. Techniques such as the Multivariate Analysis of Associations, which simultaneously examine the contribution of multiple genes is required for understanding the genetic makeup of polygenic disorders. In the future research could be also directed towards using a genome-wide association analysis and including more specific case–control study with a wider set of phenotypes.

## Competing interests

The authors declare that they have no competing interests.

## Authors’ contributions

This manuscript was prepared by AL-EITAN with support from the co-author listed. DNA extraction, SNPs selection; experimental design and statistical analysis were conducted by AL-EITAN with assistance from the co-author listed. Patient samples selection and DNA extraction were done through a collaboration link with JARADAT at Princess Haya Biotechnology Centre (PHBC). JARADAT, HULSE and TAY were assisted in designing the study to proof reading the manuscripts. All authors read and approved the final manuscript.
